# A Guided Walk through the World of Mesoporous Bioactive Glasses (MBGs): Fundamentals, Processing, and Applications

**DOI:** 10.3390/nano10122571

**Published:** 2020-12-21

**Authors:** Carla Migneco, Elisa Fiume, Enrica Verné, Francesco Baino

**Affiliations:** 1 Department of Applied Science and Technology (DISAT), Institute of Materials Physics and Engineering, Politecnico di Torino, 10129 Torino, Italy; carla.migneco@polito.it (C.M.); elisa.fiume@polito.it (E.F.); enrica.verne@polito.it (E.V.); 2 Department of Mechanical and Aerospace Engineering (DIMEAS), Politecnico di Torino, 10129 Torino, Italy

**Keywords:** bioactive glass, mesoporous, sol–gel, scaffold, porosity, bioactivity, tissue engineering, nanomaterials

## Abstract

Bioactive glasses (BGs) are traditionally known to be able to bond to living bone and stimulate bone regeneration. The production of such materials in a mesoporous form allowed scientists to dramatically expand the versatility of oxide-based glass systems as well as their applications in biomedicine. These nanostructured materials, called mesoporous bioactive glasses (MBGs), not only exhibit an ultrafast mineralization rate but can be used as vehicles for the sustained delivery of drugs, which are hosted inside the mesopores, and therapeutic ions, which are released during material dissolution in contact with biological fluids. This review paper summarizes the main strategies for the preparation of MBGs, as well as their properties and applications in the biomedical field, with an emphasis on the methodological aspects and the promise of hierarchical systems with multiscale porosity.

## 1. Introduction

Bioactive glasses (BGs) have been extensively studied for more than 50 years due to their extremely appealing characteristics, leading to an impressive increase in new application fields and, as a result, great promise in life quality and expectancy [1]. Recent studies showed that the kinetics of hydroxyapatite (HA) deposition process on the surface of BGs as well as the capability to bond to both hard and soft tissues can be improved by modifying glass surface properties [1,2]. Extensive research studies conducted on these topics have come out with a set of strategies: the possibility to get control over porosity, pore size, and pore interconnectivity, as well as to increase the external surface area of BGs ideally allow the design and development of a huge number of BG-based biomaterials with interesting textural and reactive properties [1].

In the early 1990s, an important step in BG evolution occurred when Li et al. [3] synthesized sol–gel BGs overcoming the limitations of traditional melt-derived materials [4]. Indeed, sol–gel bioactive products can be obtained in a wider range of compositions compared to the melt-derived ones. Melt-derived process needs to respect the boundaries of 60 mol.% of SiO_2_ in silicate systems because a higher amount of silica in the composition inhibits the biomaterial reactivity and apatite formation due to the more stable glassy network [5]. Owing to the larger surface area and porosity, sol–gel BGs exhibit higher bone bonding rates coupled with excellent degradation and resorption properties even using compositions with SiO_2_ content up to 90 mol.% [6].

In spite of their higher compositional versatility and bioactivity performances compared to melt-derived products, conventional sol–gel BGs are limited by the poor uniformity of the pore structure, which makes them unsuitable for some clinical applications such as controlled drug release [6,7].

At the beginning of the 21st century, a new field of applications started in the attempt to meet the surgeons’ request for having an implantable material able to effectively combat—and ideally prevent—bacterial infections, which often occur as a side effect after bone reconstruction surgery (osteomyelitis) [1]. Traditionally, patients can be treated with systemic antibiotic administration, surgical debridement, wound drainage, or even implant removal, but these techniques have important limitations and may lead to additional surgery [8].

In 2001, Vallet Regì et al. focused their attention on the possibility of using silica mesoporous materials in biomedical applications as drug delivery systems [9,10]. Ordered mesoporous silica materials were first synthesized in the 1990s by researchers from Mobil Oil Corporation and scientists from Waseda University, and their physicochemical properties were soon broadly applied in different fields, as heavy-metal adsorption, catalysis, or energy storage [10]. Vallet Regì et al. systematically investigated the properties of a set of mesoporous silica materials, including the “Mobil composition of matter” (MCM) type 41 (MCM-41) and 48 (MCM-48), hexagonal mesoporous silica (SBA-15), and phosphorous-doped MCM-41 [10,11,12]. These extensive studies showed that mesoporous silica presents unique mesoporous texture and porosity features making it an attractive material in biomedicine due to the good biocompatibility, low cytotoxicity, and huge possibilities of functionalization [10]. In spite of these appealing features, pure mesoporous silica materials suffer from poor bioactivity resulting in very slow HA deposition rates during contact with body fluids, which make them almost inadequate to be used as bioactive bone grafts [10,11,13,14].

**Figure 1:**
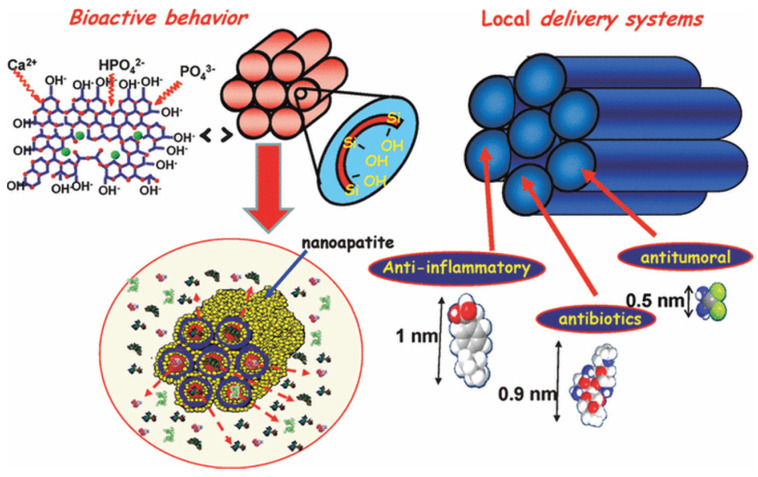
Summary of mesoporous bioactive glass (MBG) properties that make these materials highly attractive for biotechnological and biomedical applications [20].

A milestone study about mesoporous biomaterials was reported in 2004 by Yan et al., who synthesized the first mesoporous bioactive glass (MBG) combining the sol–gel method and the supramolecular chemistry of surfactants: since then, a new horizon was opened in the field of regenerative medicine [15,16,17,18]. In the last two decades, MBGs are becoming increasingly important in biomaterials research, carrying new opportunities in drug delivery, bioactive, and multifunctional systems, and overall tissue engineering applications (Figure 1) [19].

## 2. Properties of MBGs—A Short Overview

Surprisingly and in apparent contrast with Hench’s general theory about bioactivity [21], pure mesoporous silica can slowly form small amount of apatite once soaked into Simulated Body Fluid (SBF) solution in spite of the absence of Na^+^ and Ca^2+^ ions in the material composition [22]. There are multiple factors to consider for explaining this unexpected bioactive behavior [23]. One of the most reliable hypotheses individuates the most impacting factor for hydroxyapatite formation in the presence of a large amount of silanol groups (Si–OH) on the material surface [24]. Silanol groups can act as nucleation sites of the apatite layer, thus, their concentration is strictly correlated with apatite formation [23]. An apparent contradiction is that pure silica contains only Si, while crystalline apatite comprises also Ca and P. The possible explanation is that calcium phosphate deposition could come from Simulated Body Fluid (SBF) solution, which is rich in Ca^2+^ and (PO_4_)^3−^ ions and is used to test the bioactive behavior in vitro [25]. Therefore, a fundamental role in apatite development is also played by textural properties of the material. It has been demonstrated that textural features, such as pore size and pore volume are strictly correlated with apatite layer [23]. In fact, the nucleation of hydroxyapatite crystals takes place specially inside pores because of the establishment of an electrical double layer characterized by higher ionic concentration [25].

However, the mechanism behind this phenomenon is still object of study and can be partially explained by the unique textural properties of mesoporous materials, suggesting that the surface characteristics—especially the ultrahigh specific surface area—play a major role in promoting the apatite-forming ability as compared to the nominal oxide composition [15,21].

MBGs are the latest evolution of sol–gel BGs and belong to the class of mesoporous or mesostructured materials characterized by porosity in the range of 2 to 50 nm, according to the International Union of Pure and Applied Chemistry (IUPAC) nomenclature [22]. Unlike pure mesoporous silica, these biomaterials incorporate additional oxides (e.g., CaO, P_2_O_5_) and exhibit peculiar properties that make them very attractive for biomedical applications, including:

- High pore volume (about 1 cm^3^/g [10]);
- Ultrahigh specific surface area (above 100 m^2^/g [22]), which impressively accelerates the apatite-forming kinetics;
- Ordered meso-structure;
- Tunable and narrow pore size distribution (2–30 nm [10]), which can be finely controlled at the synthesis stage (e.g., by varying the type of surfactant);
- Well-defined surface properties characterized by high silanol density that allows further modifications [6,9].

In general, MBG textural properties can be properly tailored by setting different parameters during the synthesis process [26].

As previously stated, MBGs also exhibit the typical advantages of sol–gel BGs over melt-derived products, such as:

- Lower synthesis temperatures;
- Relatively easy powder technology production;
- Improved homogeneity and purity of the final products;
- Wider range of bioactive compositions even with high amount of SiO_2_ (up to 90 mol.%);
- Presence of tunable mesoporosity;
- Ability to form hierarchical scaffolds with multiscale porosity (from macro- to meso-range) [27].

## 3. Synthesis of MBGs

**Figure 2:**
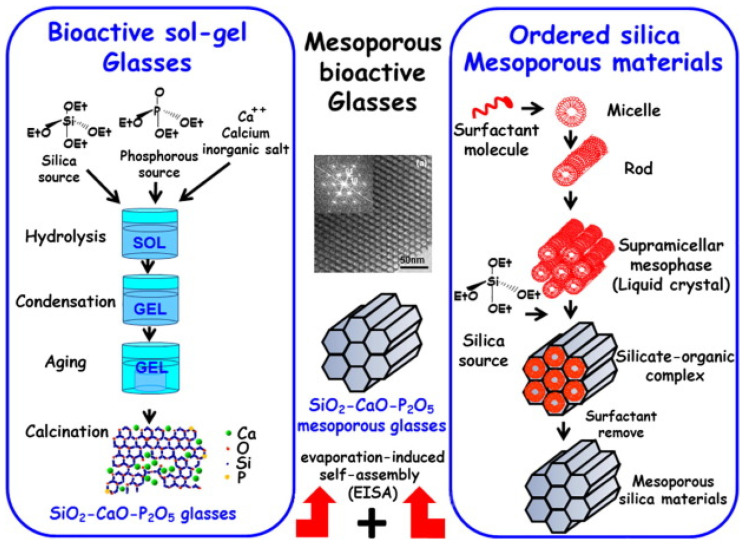
Production of MBGs by the so-called “wet” method. The image shows a gel-derived glass (**left side**) and mesoporous silica (**right side**) obtained by sol–gel and supramolecular arrangement routes, respectively. MBGs are produced by combining these two routes (middle image) [27].

The first MBG composition was synthesized by Yan et al., combining the sol–gel process with the supramolecular chemistry of surfactants (Figure 2) [6,26].

The result of this combination is a mesoporous material with composition similar to that of a conventional glass but showing the added value of an ordered mesoporous configuration [27].

According to IUPAC nomenclature, surfactants are defined as substances that lower the surface tension of the medium in which they are is dissolved and/or the interfacial tension with other phases, and, thus, they are positively adsorbed at the liquid/vapor and/or liquid/solid interfaces. [28]. In MBG synthesis, surfactant molecules incorporated into the sol act as structure-directing agents (SDA), which are essential for obtaining well-ordered structures as well as for the formation of mesopores [6,26].

Under appropriate synthesis conditions, surfactants are dissolved in a common medium (e.g., water, ethanol) with glass precursors obtaining a homogenous mixture. The surfactant molecules self-organize into micelles that are able to link with the hydrolyzed precursors (e.g., tetraethoxy sylane (TEOS) for SiO_2_ and triethyl phosphate (TEP) for P_2_O_5_), forming an ordered mesophase, where a constant ratio of network former precursor(s) and surfactant is kept [1]. The surfactant molecules link the glass precursors (mainly hydrolyzed silica resulting from TEOS hydrolysis) through the hydrophilic components, while the hydrophobic parts are kept inside the micelle interior structure [6,26].

Similarly to traditional sol–gel synthesis, the gel forms by conventional hydrolysis, condensation, and aging where micelles represent the mesostructure template [27]. After these steps, the surfactant removal may occur through calcination or extraction methods. These processes leave empty holes behind and lead to the formation of the mesoporous materials, which are characterized by a well-ordered structure with high specific surface area and porosity [14].

MBGs can be produced in different forms, including micro- and nano-sized particles [18], fibres [29], spheres [30], three-dimensional scaffolds and composites, which are all characterized by highly ordered mesoporous structure and excellent bioactivity [6,30]. The synthesis of MBG particles is the easiest preparatory method of MBG materials. They were first produced in 2004 by Yan et al., who obtained MBG particles with dimensions around several tens of nanometers [7,31]. These particles exhibited well-ordered mesoporous channels (hexagonal symmetry) of 5 nm, high surface area, and large pore volume [18]. In 2009, Lei et al. produced MBG powders [32] using acetic acid as an SDA [7]. Other variants of these methods also yielded MBG powders with excellent in vitro bioactivity [32,33,34].

MBG fibers were prepared by combining surfactant and electrospinning techniques. By an accurate control on electrospinning parameters, it was also possible to fabricate MBG fibers with hollow cores and mesoporous walls, which are highly bioactive and appealing for drug delivery applications [6,28].

MBG spheres have been synthesized by using other special methods such as alginate cross-linking, co-templating, and hydrothermal methods [30]. Yun et al. reported the production of hierarchical mesoporous–microporous MBG spheres with dimension around several hundreds of micrometers, well-interconnected pore structure, and appealing bioactivity performances [35].

From a general viewpoint, the preparative strategies for MBGs can be categorized in two main routes, which are known as the hydrothermal method and evaporation-induced self-assembly (EISA), which will be described in the following paragraphs.

### 3.1. Hydrothermal Method

The hydrothermal method was the first technique used to synthetize mesoporous silicate materials. This synthesis route improves the mesoscopic regularity of resulting products. In fact, after the solution reaction, meso-structures are subjected to re-organization and growth during the hydrothermal treatment [36].

In this method, the synthesis temperature may range from −10 to 130 °C [36]. Sometimes, the temperature of 170 °C may be reached by the introduction of a surfactant containing fluoride [36]. Higher temperatures could lead to micro-structure formation because of the degradation of ordering and decomposition of surfactants [36].

The hydrothermal procedure involves seven main steps:

1. Mixing surfactants in a solvent, typically water, at high temperature (up to 130 °C);
2. Addition of silicate precursors into the solution; hydrolysis of precursors occurs during this step through the action of an acid or base catalyst;
3. Formation of a sol composed by silicate oligomers;
4. Condensation of a gel due to interactions between silicate oligomers and surfactants and precipitation of mesoporous silicate;
5. Hydrothermal treatment, such as cooling down to room temperature, leads to sudden precipitation (solidification) of an ordered meso-structure;
6. Filtering, washing, and drying of resulted mesoporous materials;
7. Surfactant removal and consolidation by calcination to obtain the final mesoporous products [36].

Meso-structures are assembled before hydrothermal treatment, while this process is necessary to improve mesopore regularity [36]. This results in the need for a long treatment in which the hydrothermal step can last up to seven days [36].

This synthesis can be conducted under acidic or basic conditions. On the contrary, neutral conditions (pH ranging from 6.0 to 8.5) do not allow mesoporous production because polymerization and cross-linking rates of silicates inhibit the control of surfactant assembly [36].

If pH ranges between 9.5 and 12.5 (basic conditions), the polymerization and cross-linking of silicates are reversible. Acidic conditions (ranging from 1.0 to 3.0) are often preferred to synthetize mesoporous materials as they are associated to faster precipitation rates [36].

Synthesis parameters adopted in hydrothermal treatment, such as high temperature in acidic conditions and relatively slow procedures, make this treatment potentially dangerous to workers and time-consuming. For this reason, the EISA process has more recently been preferred for the synthesis of mesoporous materials.

### 3.2. Evaporation-Induced Self-Assembly (EISA)

Self-assembly is generally defined as “the spontaneous organization of materials through noncovalent interactions (such as hydrogen bond, Van der Waals forces, electrostatic forces, π–π interactions, etc.), with no external intervention [37]”. In self-assembly process, asymmetric molecules (most commonly amphiphilic surfactants) are typically engaged and pre-programmed to assemble into well-ordered supramolecular structures. After being put in contact with an aqueous solution, surfactant molecules spontaneously self-organize, exposing hydrophilic parts to the surrounding medium while shielding the hydrophobic part within a micellar interior [37].

In 1992, researchers of the Mobil Oil Company investigated surfactant self-assembly in aqueous solutions of soluble silica discovering the existence of a spontaneous co-assembly process of silica–surfactant mesophases. Since then, many research groups developed and studied mesoporous silica structures, highlighting the importance of surfactants in the synthesis process [8].

The knowledge of the chemical mechanism that regulates the behavior of surfactant/silicate solutions is key to understanding the process behind the formation of mesoporous silica and MBGs [37].

#### 3.2.1. Behavior of Surfactant Molecules in an Aqueous Solution

**Figure 3:**
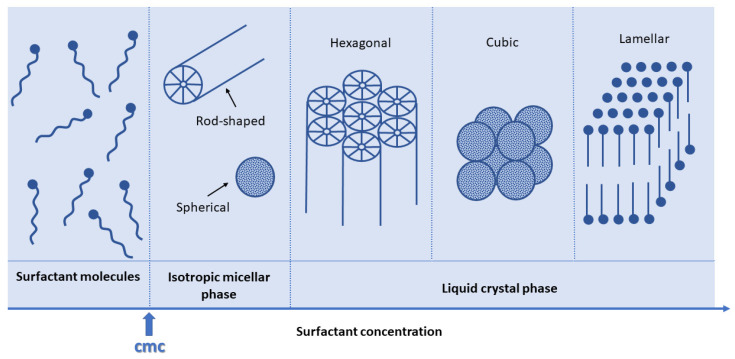
Phase sequence of a water–surfactant binary system following surfactant concentration [12].

Looking at the simple binary system represented by water and surfactant in Figure 3, SDA molecules are to be considered as very active components that can exist in different structures according to their concentration.

At low concentrations, surfactant molecules are monomolecules (i.e., are isolated) but, increasing their amount in the solution, they begin to aggregate in micelles to decrease the system entropy. The surfactant concentration at which monomolecules aggregate to form micelles has been defined as critical micellization concentration (cmc). As surfactant molecule concentration continuously grows, surfactants can exhibit different forms, such as hexagonally packed arrays, which produce hexagonal phases. Sometimes, it has been observed that surfactants can also self-organize in cubic phases, which are characterized by complex networks of rod-shaped aggregates. Thereafter, the coalescence of adjacent phases begins and the lamellar phase, which consists of mutually parallel cylinders, appears in the system [12].

The structural polymorphism exhibited by poly(ethylene oxide)-poly(propylene oxide) block copolymers had been recognized only in the 1990s [38]. Different studies had been conducted on nanostructures formed in ternary systems composed by water–alcohol as the solvent and triblock copolymer as the surfactant [38].

**Figure 4:**
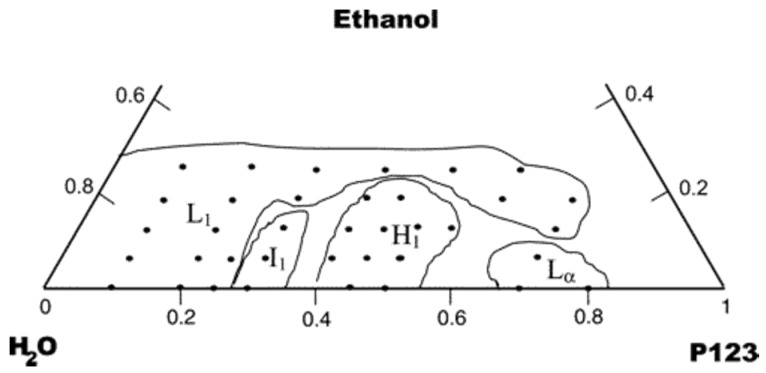
Ternary phase diagram of P123–water–ethanol system at *T* = 23 °C. L1 denotes the region with isotropic solution (water-rich), I1 refers to isotropic gels, H1 refers to cylindrical micelles arranged in a 2D hexagonal lattice, and Lα is the lamellar phase. The region boundaries are traced by solid lines [39].

**Figure 5:**
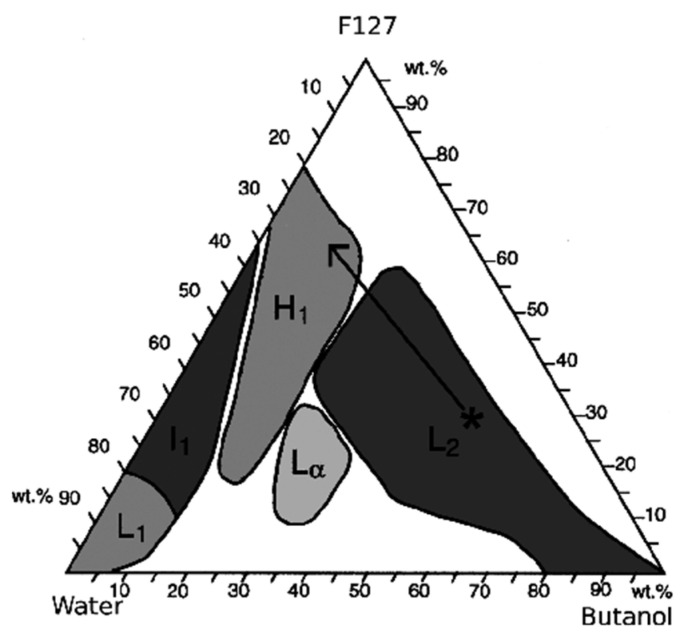
Ternary phase diagram of F127–water–buthanol system. L1 denotes the region with isotropic solution (water-rich), I1 refers to isotropic gels, H1 refers to cylindrical micelles arranged in a 2D hexagonal lattice, Lα is the lamellar phase, and L2 is a reverse isotropic micellar phase. The region boundaries are traced by solid lines. The arrow indicates the delicate balance of these regions: for example, the rapid evaporation of solvent may occur during some processes, such as spin-coating, thereby producing a transition from lamellar to hexagonal phase [38].

The presence of one of these phases rather than another in the final product mainly depends on the solvent-to-surfactant ratio, as illustrated by the ternary phase diagrams shown in Figure 4 (water–ethanol–Pluronic P123) and Figure 5 (water–buthanol–Pluronic F127). This ratio dictates the existence of a specific nanostructural arrangement of the system, which may result in mesopores with different spatial arrangement in the calcinated product (e.g., hexagonal vs. cubic symmetry) [38,39].

The obtained structure depends on the ternary system composition. A copolymer with a given block composition and molecular weight has the ability to differently swell (depending on the amount of available solvent) once it self-assembles into micelles and, in this way, it modulates the interfacial “curvature” of the resulting mesoporous structure [38,40].

The structural phase transition is mainly dependent by the length of the alkyl chain of the surfactant [41]. The study carried out by Liu et al. [41] demonstrated that an increase in surfactant chain length corresponds to structural changes, e.g., from the hexagonal mesophase, through an intermediate structure, to the cubic mesophase. Therefore, the amount of ethanol, which absolves the role of the cosolvent, impacts the pore size of mesoporous silica [41].

In MBG syntheses, the aim is to achieve structures prevalently characterized by the hexagonal phase. Indeed, packed hexagonal arrays are particularly appealing for drug delivery applications owing to their ordered structures.

#### 3.2.2. EISA Process in MBG Synthesis

**Figure 6:**
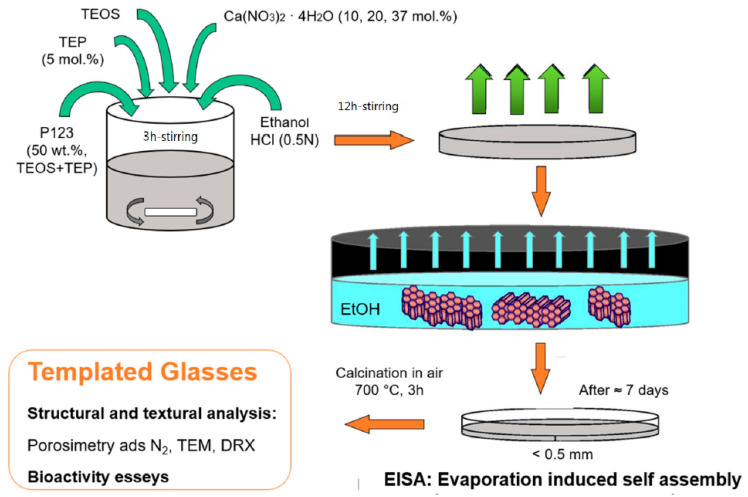
Stages of the evaporation-induced self-assembly (EISA) process for the production of MBGs. Image adapted from [14].

The synthesis batch of MBGs is a quite complex system composed by a homogenous solution of surfactant, soluble silica (from TEOS), and other ionic species (from soluble salts acting as precursors for modifier oxides in the glass) mixed in alcohol/water solvent, with an initial surfactant concentration (*c_o_*) lower than cmc. The preferential evaporation of ethanol induces the deposition of a film, formed by a non-volatile surfactant and silica/ionic species in water [37]. Then, surfactant concentration progressively increases, driving the self-assembly processes of silica–surfactant micelles organized into liquid–crystalline mesophases. The final result of this mechanism is the rapid formation of mesophase ultra-thin film well orientated according to the substrate surface [37]. The EISA process is schematically displayed in Figure 6.

The mechanism is similar to all mesoporous silicate materials: First, a diluted solution containing surfactant and precursors is prepared by mixing in alcohol (often ethanol)/water solvent [42]; at room temperature, solvent evaporation begins, increasing the surfactant concentration and, once the cmc of the surfactant molecules is reached, the self-assembly process into micelles is triggered, and further organization into liquid crystalline mesophase occurs. The “fingerprint” of EISA process is the “evaporation-induced” stage in which the template is eliminated, giving birth to the final product. After the surfactant is completely removed by thermal treatment, such as calcination, empty holes are left in the glass networks and MBGs characterized by a well-ordered structure are obtained [42].

Evaporation temperature in the EISA process is another important parameter to control in order to tailor the textural properties of MBGs. It has been demonstrated that using the same MBG composition, the mesostructure can change form 3D cubic to 2D hexagonal as a result of decrease in the solvent evaporation temperature, for example from 40 to 20 °C [42]. These two arrangements can coexist at intermediate temperatures. When solvent evaporation is conducted at higher temperatures, micelle size increases, reducing hydrogen bonds between micelles and water and leading to the formation of a 3D cubic mesostructure [42]. On the contrary, 2D hexagonal structures are obtained when the synthesis is conducted at low evaporation temperatures [42].

The textural features of MBGs are also strongly influenced by surfactant nature, such as copolymer molecular structure, and synthesis conditions during mixing, including solvent composition, temperature, and pH [26].

## 4. Effects of Surfactants and Composition

MBG synthesis involves the combination of the sol–gel process and supramolecular chemistry of surfactants. Surfactant molecules act as SDAs, which template a glass network and leave empty holes in the MBG structure once they are removed [28]. Generally, a homogenous solution of surfactant in solvent is necessary to produce ordered meso-structures [36].

Surfactants can be classified into three categories, i.e., cationic, anionic and non-ionic surfactants [36]. Cationic surfactants exhibit excellent solubility, high cmc values, and may be used in both acidic and basic media [36]. On the other hand, they are often toxic and expensive [36]. Anionic salt surfactants include carboxylates, sulfates, sulfonates, phosphates, etc. Non-ionic surfactants, such as P123, are also widely available in different chemical structures [36]. They are typically non-toxic, available at an affordable cost, and biodegradable. Therefore, they are widely employed for industrial applications [36].

### 4.1. Brief Overview of Surfactants Used in the Production of Mesoporous Materials

The specific phase existing in a surfactant-containing sol at a given concentration depends not only on the template concentration but also on its properties, i.e., the length of the hydrophobic carbon chain, hydrophilic head group, and counterion [12].

The most commonly used surfactants in MBG synthesis are cetyltrimethylammonium bromide (CTAB), Pluronic^®^ P123, and Pluronic^®^ F127.

#### 4.1.1. CTAB

CTAB (C_16_H_33_N(CH_3_)_3_Br), molecular weight 364.45 g/mol) is a quaternary ammonium surfactant that is one of the main components of some buffers for the extraction of DNA. CTAB is widely used in the synthesis of gold nanoparticles (spheres and rods) and mesoporous silicate materials [43]. As other surfactants, CTAB forms micelles in aqueous solutions when a temperature of 30 °C is reached. The formed micelles have an aggregation number between 75 and 120 and a degree of ionization within 0.2–0.1 [43].

For these properties, CTAB has been the first template used for the synthesis of mesoporous materials. The products of sol–gel synthesis combined with CTAB possess regular arrays of uniform channels, the dimensions of which can be tailored tuning different factors, as the choice of surfactant, auxiliary chemicals, and reaction conditions [43].

#### 4.1.2. Pluronics^®^

This class of surfactants is composed by poly(ethylene glycol)–poly(propylene glycol)–poly(ethylene glycol) (PEO-PPO-PEO) triblock synthetic copolymers that are thermos-reversible in aqueous solutions [44]. Their sol–gel transition is influenced by the composition, molecular weight, and concentration of each constituent block polymer [45].

Pluronics have an amphiphilic structure in which ethylene oxide constitutes the hydrophilic part, while propylene oxide is the hydrophobic component [44]. Indeed, this class of triblock polymers is composed by a polar, water-soluble group attached to a non-polar, water-insoluble hydrocarbon chain [46].

These surfactants self-assemble into amphiphilic micelles that are able to accommodate lipophilic molecules in the central core area [47]. Owing to this peculiarity, Pluronic micelles are effectively employed as drug carriers because their assemblies can be used as passive drug containers.

Pluronic micelles also have other appealing features such as low in vivo toxicity and an appropriate size that reduce renal excretion [47]. The aggregation state of these micellar systems can be tuned just selecting the appropriate Pluronic size and PPO/PEO block–length ratio [47].

Pluronic^®^ P123 is a symmetric triblock copolymer formed by PEO and PPO in an alternated linear chain according to the sequence PEO_20_–PPO_70_–PEO_20_. The peculiar characteristic of this surfactant is influenced by the PPO unit, which is hydrophobic above 15 °C and soluble in water below 15 °C, leading to the formation of micelles made up of a hydrophobic core of PPO block and a hydrophilic corona of PEO groups [39].

In acidic conditions, P123 is cleavable [44], thereby allowing the interaction of the terminal hydroxyl groups of the PEO block with TEOS [44].

Pluronic^®^ F-127 (block sequence PEO_16_–PPO_70_–PEO_16_), also known as Poloxamer 407, is a non-ionic surfactant polyol that has been often employed in tissue engineering because of its sol–gel transition near the physiological temperature and pH [48]. Its mechanism of action is analogous to that of P123.

### 4.2. Surfactant Molecules as Structure-Directing Agents in MBG Synthesis

**Figure 7:**
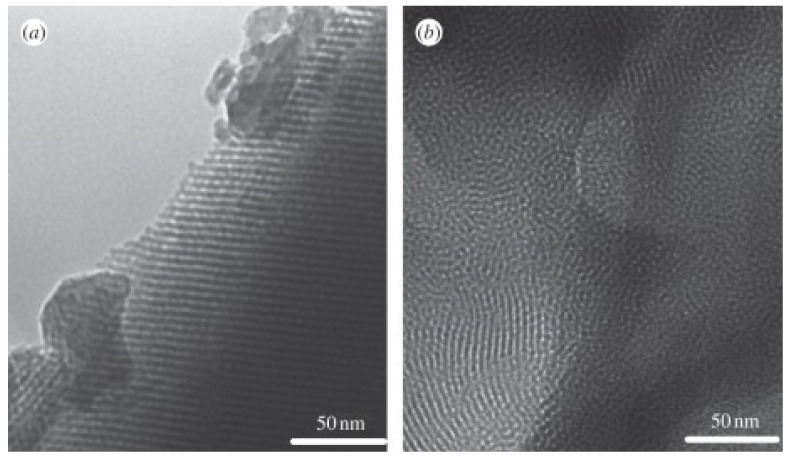
Transmission electron microscopy images of (**a**) P123-templated MBG and (**b**) cetyltrimethylammonium bromide (CTAB)-templated MBG [7].

**Figure 8:**
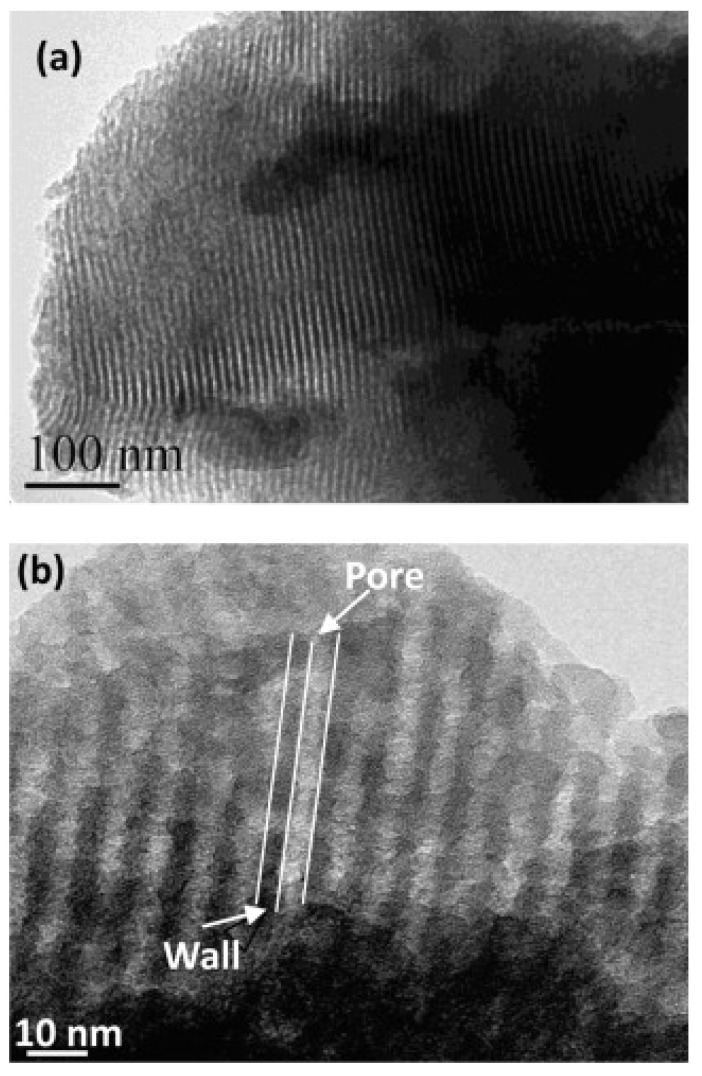
Typical TEM images of F127-induced MBGs (**a**), in which the pore and wall structures are indicated by white lines in (**b**) [49].

It has been demonstrated that the choice of surfactant directly impacts on mesopore structure, mesopore size, and pore volume of MBGs [7]. A lot of studies have been conducted on the main SDAs used for MBG synthesis, such as CTAB, F127, and P123 (Figure 7 and Figure 8) [7]; the resulting MBGs exhibit different final mesopore size, volume, and surface area (Table 1), ranging from 2 to 10 nm, 0.4 to 0.7 cm^3^, and 150 to 1000 m^2^/g [27].

It has been generally noticed that P123 induces a more ordered nano-architecture on the final MBG product than CTAB, as shown in Figure 7.

Mesopore size is also different in CTAB-templated MBGs as compared to MBGs produced by using Pluronics: in fact, CTAB-derived materials are typically characterized by lower pore size (2–3 nm) than P123- or F127-based systems (4–10 nm) [7]. Furthermore, SDA choice strongly affects pore architecture: P123 and CTAB usually induces a 2D hexagonal mesopore structure, while F127 induces a wormlike structure; furthermore, the order in CTAB-induced MBGs is lower than that in Pluronic-templated ones [7].

Considering pore volume, P123-templated MBGs exhibit higher pore volume and specific surface area than F127 ones. This feature is reflected on a significantly higher drug loading efficiency of P123-based MBGs compared to F127-based MBGs with the same composition [7]. Arcos et al. have also demonstrated that MBGs synthesized by using CTAB have higher drug loading efficiency (10.7%) compared to the same MBG composition deriving from P123 and F127 (9.7% and 9.1%, respectively) [50]. This could be explained by the better dimensional matching between CTAB-derived mesopores and small drug molecules. For this reason, the usage of CTAB is recommended to improve drug delivery performances of MBGs [6,50].

On the other hand, the use of Pluronics carries some advantages from a preparative viewpoint: in fact, the employment of P123 and F127 is considered much easier than that of CTAB because of its need of additional filtering and washing procedures for obtaining a material suitable to be calcinated [7].

### 4.3. Composition–Mesopore Structure Relationship in MBGs

Although pure mesoporous silica exhibits excellent textural properties and meso-scale order, it lacks in bioactivity behavior due to its composition (100% silica). Pure SiO_2_ architecture is characterized by an extremely rigid network that is not able to easily interact with the physiological environment, thus inhibiting the dissolution process behind the bioactivity mechanism.

In order to overcome this limitation, a huge number of multicomponent MBG systems have been developed over the last two decades.

**Figure 9:**
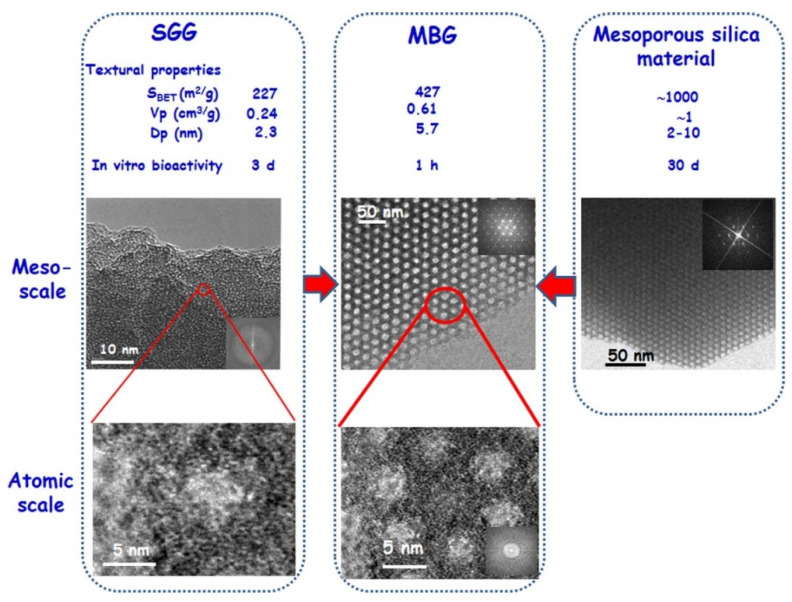
High-resolution transmission electron microscopy images and electron diffraction patterns of conventional sol–gel SiO_2_–CaO–P_2_O_5_ glasses, SiO_2_–CaO–P_2_O_5_ MBG, and pure mesoporous silica [42].

Generally, the higher the content of silica in MBG composition, the higher the order of mesopore arrangement along with the higher pore volume and specific surface area [7]. As schematically shown in Figure 9, MBGs exhibit intermediate textural properties between conventional sol–gel BGs (without the use of SDAs) and pure mesoporous silica [42]. Textural features of MBGs, such as pore volume and specific surface area, are almost twice than those of gel-derived glasses with the similar composition [42]. As a result of the higher surface area available for ion-exchange reactions with biological fluids, the apatite-forming rate of MBGs is dramatically higher than that of conventional sol–gel glasses (1 h vs. 3 days, see Figure 9) [51].

Researchers have mainly focused their attention on MBGs based on binary (SiO_2_–CaO) or ternary (SiO_2_–CaO–P_2_O_5_) multicomponent systems, and only recently have quaternary MBGs including metallic dopants been investigated [7].

The most commonly used precursor for SiO_2_ is TEOS; tetramethoxy silane (TMOS) was also used to obtain mesoporous spheres with very narrow size distribution (monodisperse systems) [52], but its toxicity limits its widespread use.

#### 4.3.1. Role of Calcium Oxide

The introduction of CaO (typical precursor: calcium nitrate decahydrate) into pure mesoporous silica strongly impacts on the mesoporosity of the silicate walls and the overall mesoporous structure [42].

By changing the CaO amount in glass composition, different mesostructures can be obtained, such as 3D cubic or 2D hexagonal arrangements [42].

CaO is a modifier oxide that disrupts the network glass structure decreasing the network connectivity. Thus, the increase in Ca^2+^ content is directly related to the increase of inorganic/organic volume ratio of micelles, which decreases the curvature of surfactant micelles, contributing to the formation of hexagonal phases [42]. On the contrary, three-dimensional cubic arrangement is shown when CaO content in MBGs is lower.

Although a decrease in textural properties (surface area and pore volume) and the introduction of potential structural defects have been observed with the incorporation of CaO, MBGs still possess a high surface area and pore volume (see Figure 9 and Table 2) [53].

Ca^2+^ ions are also responsible for the excellent and extremely quick in vitro bioactivity behavior exhibited by MBG compositions once in contact with simulated physiological fluids [42]. This occurs because Ca^2+^ presence on the glass surface decreases locally the pH of the solution to a value around 6.7, which allows the formation of octa-calcium phosphate, which is an intermediate phase for the formation of nanocrystalline HA, thus accelerating the bone mineralization process [42].

Other studies have shown that the increased bioactivity related to CaO content leads to enhance drug loading efficiency while decreasing drug release rate and burst effect, thus playing an important role in modulating the drug delivery kinetics [33]. The CaO influence on drug release ability can be explained by drug chelation with Ca^2+^ ions on the pore walls, thus inhibiting the release process [9].

#### 4.3.2. Role of Phosphorous Oxide

The presence of P_2_O_5_ is not strictly essential either for the formation of MBGs or the bioactive properties (CaO may be enough in this regard), but it is somehow beneficial in concentration around 5 mol.% as far as the mesopore structural design is concerned. In fact, the presence of phosphorous atoms in the glass composition induces the formation of 3D cubic arrangements in MBGs, while a 2D hexagonal nanostructure is naturally obtained in P-free compositions [42]. This is due to the fact that P_2_O_5_ spontaneously binds to CaO, leading the formation of amorphous calcium phosphate clusters on the glass surface; hence, calcium ions are ejected from the glass network, affecting the structure similarly to a decrease in CaO amount, thus facilitating the cubic structure formation [42].

TEP is typically used as P_2_O_5_ precursor; other options such as phosphoric acid are discouraged as they are typically associated to a significant decrease of specific surface area and mesopore volume (see Table 2).

#### 4.3.3. Role of Dopants

Although Si, Ca, and P are the main elements of MBG compositions, small amounts of other components (typically modifiers) have been recently introduced into MBG systems in order to impart special extra-functionalities. In fact, a number of metallic cations are known to play therapeutic actions once released in vitro and in vivo, stimulating beneficial biological responses such as angiogenesis (e.g., copper and cobalt ions) and antibacterial effect (copper and silver ions).

In general, the introduction of divalent ions (e.g., Mg^2+^, Zn^2+^, Cu^2+^, or Sr^2+^), trivalent ions (Ce^3+^, Ga^3+^, or B^3+^) or tetravalent ions (Zr^4+^) into the parent SiO_2_–CaO–P_2_O_5_ system leads to a decrease of specific surface area and pore volume in the final MBG (Table 2). In fact, the incorporation of dopants has a negative effect on the precursor condensation, disrupting the ordered orientation of silicate [(SiO_4_)^4^^-^] units during the self-assembling reaction.

#### 4.3.4. The Challenge of Multicomponent Mesoporous Systems

A review of the literature shows that most MBGs belong to binary (SiO_2_–CaO) or ternary (SiO_2_–CaO–P_2_O_5_) systems doped with small amount of trace elements, which usually act as network modifiers [53,54,55,56,57,58,59,60].

A great technological challenge in the field of MBGs is the synthesis of multicomponent complex systems constituted by more oxides (e.g., five or six oxides [61]) in similar proportions, and there is a relative paucity of literature on this topic. Shoaib et al. [62] have recently published a work describing a MBG composition belonging to the five-oxide 49SiO_2_–20CaO–20Na_2_O–7K_2_O–4P_2_O_5_ (mol.%) system. Fiume et al. have deeply investigated the impact of different processing methods (i.e., melt-quenching route vs. sol–gel synthesis) on the textural properties and bioactivity of a six-oxide SiO_2_–CaO–K_2_O–P_2_O_5_–Na_2_O–MgO glass, confirming the key role played by mesopores in accelerating the HA-forming kinetics [61].

During the synthesis of MBG multicomponent systems, there are mutual interactions among oxide precursors that can also interact with the supramolecular chemistry of surfactant according to ion-recombination mechanisms, which are difficult to predict. Indeed, the synthesis of mesoporous products based on such complex systems may suffer from solubility problems since the earlier synthesis stages. Therefore, the formation of micelles depends on PPO and PEO block solubility, which is dependent on the solvent used and the temperature [63]. The dissolution of several commercial surfactants in pure water is quite difficult at low temperature and not recommended, while several precursors have very low solubility limits in alcoholic solutions and need higher temperatures. Furthermore, the synthesis optimization should properly consider the solvent-to-surfactant ratio in order to achieve cylindrical micelles arranged in a 2D hexagonal lattice.

For all these reasons, the production of multicomponent mesoporous systems is still a challenging and appealing issue that may open new frontiers to further clinical applications.

## 5. Biomedical Applications of MBGs

### 5.1. Bone Regeneration

The intrinsic characteristics of MBG materials indicate them as appealing biomaterials for bone tissue engineering applications [7]. In the last decade, a huge number of studies have been carried out to evaluate the in vitro bioactive potential and reaction kinetics of different MBG-based products in contact with body fluids. Apatite formation on the surface of MBG particles and scaffolds was first studied by Yan et al. [18] who reported HA presence in vitro after only 4 h of immersion in SBF. The extraordinary ability of MBGs to induce apatite mineralization significantly faster than conventional melt-derived and sol–gel BGs is the result of the high surface area and pore volume, which strongly enhance the surface reactivity of the material due to an increased number of exposed silanol groups (Si-OH) at the interface with the external environment [6,17,22,23,24].

**Figure 10:**
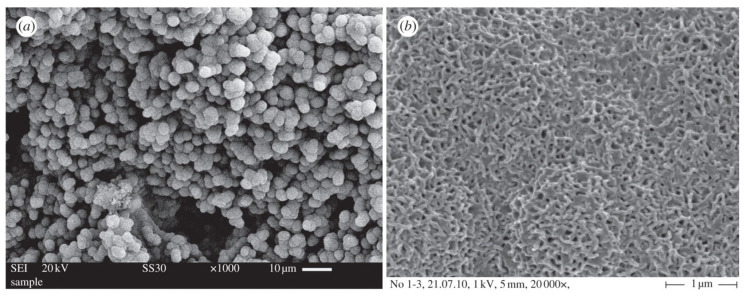
Nano-crystalline apatite mineralization on the surface of three-dimensional MBG scaffolds: (**a**) “cauliflower” globular agglomerates (low magnification image), (**b**) details of nano-crystals (high magnification image) [7].

Other studies conducted by Garcia et al. investigated the mechanism of apatite mineralization through nuclear magnetic resonance (Figure 10) [64]. This analysis confirmed the significant difference on apatite-forming kinetics between non-mesoporous BGs and MBGs. In conventional BGs, apatite formation may occur over a time period ranging from a few hours to 1–2 days through three major subsequent steps in which (i) the glass releases cations and (ii) forms Si–OH that (iii) then form networks by repolymerization [7]. On the contrary, the MBG surface appears “just ready” to execute these three steps faster, accelerating and increasing the whole process of HA mineralization (<8 h) [64].

### 5.2. Drug Delivery Systems

In the 21st century, the new trend of pharmaceutical companies has progressively changed from focusing the attention only on the design and synthesis of new drugs to a combined strategy of developing both new drugs and innovative drug delivery systems (i.e., platforms for their release). Most advanced strategies for the treatment of major disease are all based on the use and delivery of macromolecular drugs [65]. For example, immunization therapies for protection against many infectious diseases, such as AIDS, are related to delivery of protein vaccines [66]. The delivery of gene compounds is also fundamental for all gene therapies adopted for the treatment of carcinoma and haemophilia, etc. [67,68].

The delivery of proteins and cells also plays an important role in regenerative medicine for the repair and replacement of organs and tissues [65]. In this regard, osteomyelitis incidence is one of the most important causes of surgery failure, and traditional techniques are applied just to prevent further complications once the infection is already underway. Current treatments such as systemic antibiotic administration, surgical debridement, wound drainage, and implant removal may cause extra traumas to patients. Furthermore, conventional drug administration, i.e., intravenous or intramuscular injections and oral pills, shows variable drug concentration during the assumption reaching toxic peaks and declining rapidly to ineffective values [2,32,69]. Hence, continuous evolutions and improvements are brought in this research area in the attempt to develop truly functional systems with high drug delivery efficacy and reduced toxicity [1].

Controlled drug release has gained increasing attention aiming at the development of targeted sustained drug delivery systems able to release effective drug concentration into injured sites while limiting all side effects associated to conventional drug administration.

**Figure 11:**
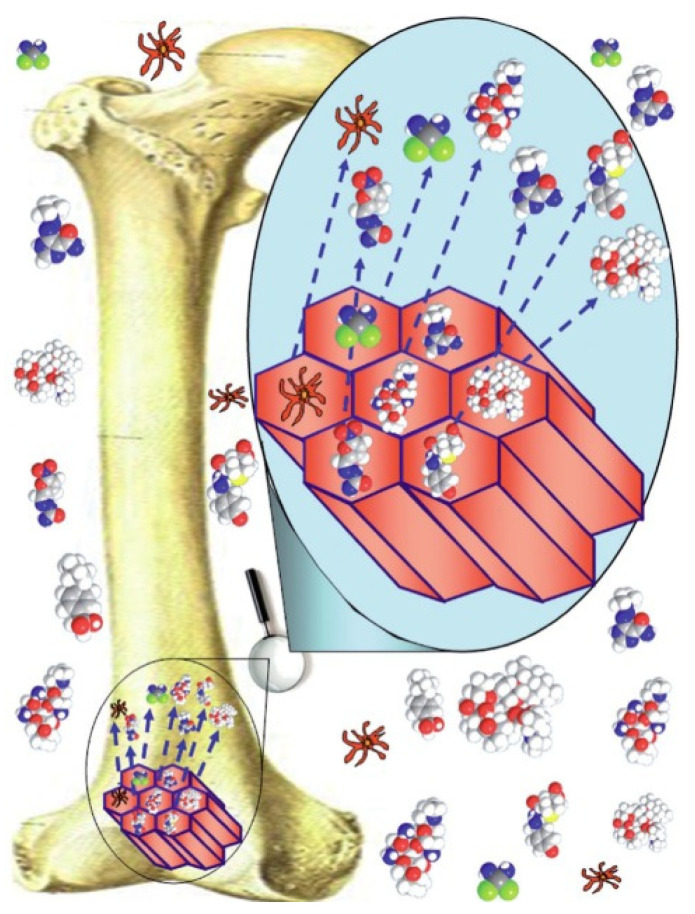
Schematic concept of using MBGs for drug delivery and bone regeneration [70].

In this regard, one of the most appealing features of MBGs is their very high surface area and pore volume. These two characteristics are strictly correlated with the loading efficiency for drugs and growth factors in the material. For this reason, MBG-based devices are considered highly attractive platforms for controlled drug delivery (Figure 11) [7].

Drug-loading efficiency and release kinetics strictly depend on environmental conditions but also on mesopore characteristics. MBGs can be finely tailored by acting on the synthesis parameters and are suitable as carriers for a range of drug molecules [27].

The high density of Si–OH groups on the MBG surface plays a fundamental role in the interactions with drugs and protein, exhibiting an appealing mechanism of slow and sustained drug release kinetics [33].

Extensive researchers have demonstrated that compared to non-mesoporous BGs, drug release in MBGs is more efficient (Table 3) because of the Fickian diffusion mechanism that regulates MBG dissolution in aqueous solutions [71]. Furthermore, it was also reported that the apatite formation on the MBG surface may improve drug-loading efficiency and decrease burst release and release rate due to drug chelation effect by calcium ions, as already discussed in Section 4.3.1 [29,72].

### 5.3. MBGs as Multifunctional Platforms for Tissue Repair

Recent studies have investigated the role of MBGs as multifunctional platforms that can absolve to different functions through the release of therapeutic ions and/or drug/growth factors [69]. In this regard, researchers recently found in MBGs loaded with dimethyloxally glycine (commonly used as neuroprotective agent) an increased osteogenic and angiogenic activity of stem cells, while doxorubicin-loaded MBGs (doxorubicin is an antibiotic and anthraclyne drug) inhibited the viability of cancer cells [69].

The therapeutic effect of released drugs can be synergistically combined with that of ion dissolution products from the glass, which are known to stimulate osteogenic differentiation and bone tissue regeneration [74].

Furthermore, antibacterial effects, stimulation of cementogenesis in dental applications, and angiogenesis are all demonstrated effects carried by these extremely appealing biomaterials through the release of therapeutic cations (Table 4).

## 6. The Last Frontier: Hierarchical MBG Scaffolds

### 6.1. Fabrication of MBG Scaffolds: From Macro- to Meso-Scale… and Back

The fabrication of hierarchical MBG-based scaffolds with multiscale porosity is one of the most attractive challenges in modern biomaterial science and biomedicine. In fact, mesopore size is almost three orders of magnitude lower than that of human cells, such as osteoblasts, and for this reason, macroporosity should somehow be introduced in the final device with the aim to allow cell colonization and tissue regeneration [80].

Three-dimensional porous scaffolds based on MBG materials are typically produced by the combination of SDAs with other techniques [7]. The first MBG scaffold was fabricated in 2008 by Yun et al. [81], who used methylcellulose as a sacrificial porogen to create hierarchical structures characterized by large-size macropores (about 100 μm) and mesopores at the nanoscale.

**Figure 12:**
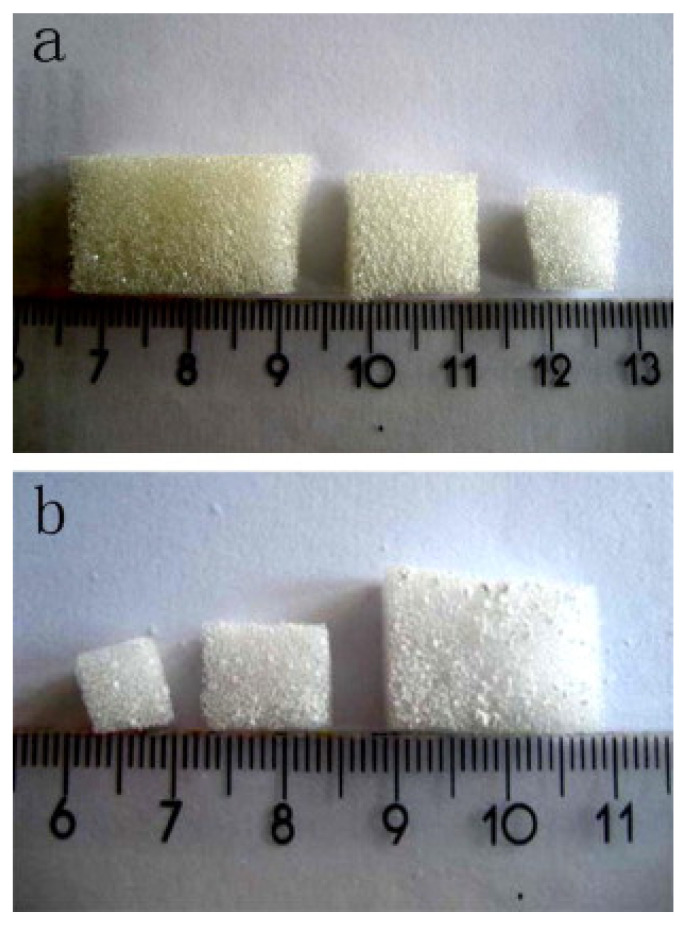
Photographs of (**a**) the polyurethane sponges used as macroporous templates and (**b**) the resulting hierarchical MBG scaffolds [56].

**Figure 13:**
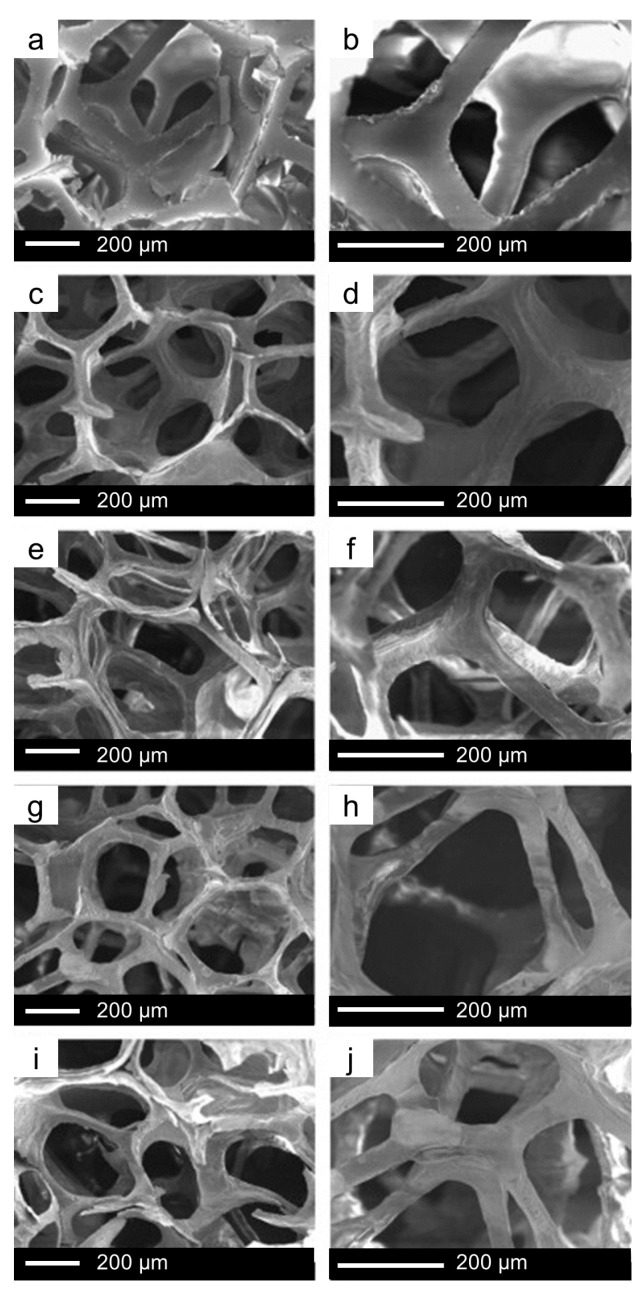
SEM analysis of polyurethane sponges (**a**,**b**) and macro-mesoporous scaffolds with composition (mol.%) 100SiO_2_ (**c**,**d**), 90SiO_2_–5CaO–5P_2_O_5_ (**e**,**f**), 80SiO_2_–15CaO–5P_2_O_5_ (**g**,**h**), and 70SiO_2_–25CaO–5P_2_O_5_ (**i**,**j**) [56].

Another widely used method is the polymer co-templating technique, which consists of the combined usage of SDA and macroporous template. In 2008, Wu et al. successfully fabricated hierarchically porous three-dimensional MBG scaffolds based on different glass compositions by using a commercial polyurethane sponge as a template for the interconnected macroporous structure and P123 as an SDA for the synthesis of MBG (Figure 12 and Figure 13) [56].

Li et al. [82] also prepared MBG scaffolds by using a similar method, i.e., combining polyurethane foam, responsible for the final macroporosity, and P123 surfactant, as a template for mesoporosity [82]. The glass foams exhibited a hierarchical structure characterized by interconnected macropores (about 200–400 or 500–700 μm), which allows in vivo cell colonization and tissue regeneration [82].

Both these studies reported excellent results in terms of bioactive performance during immersion in vitro in simulated body fluid.

The general advantages in the use of the polymeric sponge template method include the high interconnectivity of macropores, the tunable macropore size of the polymer, and the inexpensiveness of the technique. However, the mechanical properties of foam-replicated MBG scaffolds remain quite low even after a surface reinforcement with silk fibroin coating (50 to 250 kPa [72]) as compared to cancellous bone (2–12 MPa [83]).

Similar results were obtained by using natural templates, such as cattail stem [84] or mushroom talk [85], instead of the synthetic polymer sponge.

Another important method for the fabrication of MBG-derived scaffolds is three-dimensional plotting, also called direct writing or three-dimensional printing, which allows a better control on pore morphology and size to be achieved [7]. In this technique, the product structure is built according to a layer-by-layer approach during the plot under mild conditions. Both Yun et al. [86] and Garcia et al. [55] fabricated a hierarchical three-dimensional porous MBG scaffold by using a combination of double-polymer templating and rapid prototyping. In these experiments, MBG gel was mixed with methylcellulose; then, the resulted viscous solution was printed in the desired structure and finally sintered at 500–700 °C to remove polymer templates and obtain the final MBG scaffolds [6,87]. These experiments yielded MBG scaffolds with uniform pore structure but poor mechanical strength owing to the presence of micropores in the solid structure due to the use of methylcellulose. The main disadvantages of this method include the need for methylcellulose and an additional sintering procedure. For this reason, a variant of three-dimensional printing was proposed substituting methylcellulose with poly(vinyl alcohol). The MBG scaffolds resulting from this method exhibited good mechanical strength (200 times higher than MBG scaffolds obtained by methylcellulose) and mineralization ability in simulated body fluids, which make them highly promising for bone regeneration applications [42].

The polymer used to prepare the ink for printing may also “survive” in the final scaffold, avoiding an additional thermal treatment and leading to the development of hierarchically porous composites. Yun et al. [88] combined three-dimensional printing with salt leaching to produce polycaprolactone (PCL)/MBG composite scaffolds with three levels of porosity, including mesopores of 5 nm (inherent of solid MBG walls), small macropores from 2 to 9 μm (left behind by small salt crystals), and giant regular macropores (size around 200 μm) deriving from the printing process. These scaffolds were fabricated by direct extrusion of the composite ink onto a platform through a nozzle of 500 μm and exhibited suitable compressive strength (2–4 MPa) for bone repair, fast apatite-forming ability in vitro, and good pliability, suggesting a possible use in osteochondral tissue engineering.

A comprehensive overview of three-dimensional printing applied to bioceramics and BGs, including MBGs, has been recently provided elsewhere [89].

### 6.2. The Potential of MBG Scaffolds for Advanced Therapies

Over the last decade, a lot of experiments have been carried out about the multifunctional ability of ion-doped MBG scaffolds, pushing their potential beyond “traditional” bioactivity and opening a new frontier in regenerative medicine [90].

Among monovalent ions, silver and lithium have shown promise for doping biomaterials to develop innovative clinical applications [91]. Silver has the highest level of antibacterial activity among all the heavy metals that are employed for this purpose [27]. The antibacterial effect of silver is produced because Ag^+^ ions strongly interact with disulfide (S–S) and sulfhydryl (–SH) groups exposed on microbial cells surface, thus leading to the formation of S–Ag bonds [27]. Once the silver ions are bonded onto the surface proteins of microbial walls, they inhibit the respiration process of bacteria, triggering the cascade of rescue mechanisms that ultimately lead to bacterial cell death [27]. Ag^+^ ions absolve their antibacterial role also causing the proton leakage in the bacterial membrane, thus leading to its disruption and allowing Ag^+^ ions to enter cell cytoplasm where they can provoke irreversible conformational changes and apoptosis [26,90].

Several experiments conducted about Ag-doped MBG scaffolds have shown how the introduction of this element cause some changes in structural, morphological, and textural properties compared to Ag-free MBGs [27,92]. For example, Vulpoi et al. reported a progressive decrease in surface area and change in mesoporous features with the increasing content of silver in the MBG composition [93]. This is consistent with the general role played by modifiers in disrupting the meso-structural order in MBGs.

The antibacterial effects of Ag-doped MBG scaffolds have been deeply analyzed by Zhu et al., who showed a strong dependence on glass functionalization with γ-aminopropyl triethoxysilane, which improved both antibacterial effect and drug-loading capability Yunof Ag-doped scaffolds [94].

Lithium has been widely employed over the past 50 years in the treatment of depressive disorders, playing a role in mood stabilization [89,93]. It is thought that the beneficial neural effects of lithium are due to the activation of the Wnt signaling pathway, which enhances the re-myelinization of peripheral nerves and increases the proliferation of neuronal progenitor cells [95]. Lithium has been recently studied for its effects on bone density, and it was found that this trace element interferes with calcium transport and improves the proliferation and differentiation of osteoblasts as well as cementogenic gene expression in periodontal cells [69,89,94,95]. In this regard, Han et al. [76] produced Li-doped foam-replicated MBG scaffolds for possible use in the treatment of periodontal disease. These hierarchical scaffolds still maintained mesoporous features with well-ordered and uniform nano-channels with a diameter of 5 nm. Comparing different Li-doped scaffolds to Li-free counterparts, both the number of periodontal cells attached to scaffold walls, and the cell proliferation rate was higher in MBGs with the highest Li content (5 mol.%), and a clear stimulatory effect of this element toward cementogenesis was observed as well [76,96].

Divalent ions, such as Mg^2+^, Zn^2+^, and Sr^2+^, have also been incorporated in MBG-based scaffolds to enhance bone regeneration potential [69,78,97]. Magnesium absolves an essential role in bone metabolism processes, and its lack is correlated to resorption phenomenon in bone tissue [98]. Zinc is one of the most fundamental trace elements in human organism, being involved as an enzymatic co-factor in more than 300 enzymatic reactions [99]. Strontium is naturally present in the liver, muscles, and body fluids but its highest amount is found bone tissue, being related to bone remodeling (anti-resorption properties) [77]. Wang and co-workers successfully synthesized Mg-, Zn-, and Sr-doped MBG scaffolds using Pluronic P123 and polyurethane sponge as co-templates for mesopores and macropores, respectively [78]. The calcinated glass scaffolds showed no apparent differences in terms of microstructure (no crystallization), macroporous structure, or pore volume as compared to the ion-free control [78]. Furthermore, no cytotoxic effects were elicited by Mg-, Zn-, and Sr-doped MBG scaffolds, but the release of these therapeutic ions into the culture medium contributed to enhance the proliferation of mesenchymal stem cells (MSCs) and alkaline phosphatase (ALP) activity, suggesting an improvement in bone regenerative capabilities [78].

Cu^2+^ has also been employed for doping MBG-derived scaffolds as a pro-angiogenic agent. In vitro culture showed that Cu^2+^ ions enhanced the proliferation of endothelial cells in a dose-dependent manner [100], and in vivo experiments confirmed the role of Cu^2+^ ions in regulating the expression of vascular endothelial growth factor (VEGF) [79]. Pro-angiogenic properties of copper are related to its hypoxia-mimicking ability; furthermore, Cu^2+^ ions can also elicit an antibacterial effect and improve osteogenic differentiation and bone-related gene expression, thus being useful to exert a multifunctional action [79]. Cu-doped MBG scaffolds showed lower specific surface area, pore volume, and pore size as compared to the Cu-free MBG counterparts, as already observed for other ion-doped glasses; however, the value of these textural properties is still sufficient to ensure good bioactivity and sustained drug release [79].

Although MBGs are non-magnetic materials, the introduction of iron (Fe) in their composition can add magnetic properties to MBG-derived scaffolds, which could be exploited for cancer treatment by hyperthermia [101]. Wu et al. doped foam-replicated MBG scaffolds with 5 and 10 mol.% of Fe and reported that the order of mesopore arrangement decreased as the iron content increased. In fact, 5Fe-MBG scaffolds maintained an ordered hexagonal structure with mesopore size of 5 nm, while 10Fe-MBG scaffolds showed a lack of order in mesopore distribution [53]. However, the surface area was high, mesopore size was not affected by the iron doping, and the apatite-forming kinetics in vitro were comparable to those of the Fe-free MBG system. Being magnetic and highly bioactive, Fe-doped MBG scaffolds are promising candidates as a multifunctional platform addressing several functions for diseased bone, including bone regeneration, anticancer drug delivery, and hyperthermic treatments [53].

## 7. Outlook

Since their invention in 2004, MBGs have convincingly proved to be highly versatile biomedical platforms for stimulating bone regeneration and supporting sustained drug delivery. Specifically, the appealing features of MBGs also offer the opportunity to achieve attractive results in terms of finely controlled drug release. Indeed, mesopore channels may be imaged as drug reservoirs that can be opened and closed on demand in response to external stimuli, such as pH, temperature, etc. [102]. The possibility of designing and developing “smart” materials able to establish a deep interaction with the biological environment to precisely control the release process of therapeutical factors has opened a new attractive and unexplored field for researchers all over the world.

Furthermore, MBGs are suitable to elicit a set of additional extra-functionalities after being doped with specific therapeutic ions, such as angiogenesis (e.g., doping with copper or cobalt), antibacterial effect (e.g., doping with silver or copper), and magnetic hyperthermia (e.g., doping with iron).

The fabrication of MBG-based hierarchical systems with multiscale porosity also is highly attractive. Although macro-mesoporous scaffolds produced by conventional fabrication methods still exhibit poor mechanical strength, this drawback can be successfully overcome if three-dimensional printing is applied to process MBGs, alone or in combination with polymers to produce composites.

The long time required for MBG synthesis indeed is an issue deserving further optimization in the future, as well as the challenge of producing multicomponent systems (number of oxides >4) with an ordered mesoporous texture still remains open. In fact, the higher the compositional complexity, the higher the disrupting effect on the aggregation of silicate units to form mesophases, and the higher the unwanted interactions among precursors (e.g., precipitation of insoluble salts, phase segregation).

Lastly, the development of MBGs incorporating other network formers than silica could further improve the versatility and therapeutic potential of these materials. To the best of the authors’ knowledge, only one study was reported on the synthesis of borosilicate MBGs [103], which could have appealing implications in the context of wound healing and soft tissue engineering similar to their melt-derived counterparts (e.g., 13-93B3 glass [104]).

## Figures and Tables

**Table 1:** Textural characteristics of MBGs prepared by using different structure-directing agents [7].

Structure-Directing Agent	Specific Surface Area (m^2^/g)	Pore Volume (cm^3^/g)	Pore Size (nm)
P123	300–350	0.4–0.49	4.3–4.6
	278–400	0.54–0.73	6.5–6.9
	250–350	0.4–0.5	5
	438–466		3.5–3.7
	450–480	0.63–0.73	5.37–6.43
	499	0.7	6.1
F127	520	0.51	5.4
	228–300	0.36–0.42	5.0–7.1
	152–310	0.235–0.356	4.2–5.0
CTAB	1040	1.54	1.82–2.2
	443	0.57	2.9
P123 + CTAB	552–618	0.69–1.08	4.1–6.2

**Table 2:** The effect of composition on the characteristics of mesopore structures [7].

MBGs with Different Compositions (mol.%)	Specific Surface Area (m^2^/g)	Pore Volume (cm^3^/g)	Pore Size (nm)	References
100Si	490		3.6	[54]
95Si5Ca	467		3.7	
90Si10Ca	438		3.5	
100Si	310	0.356	4.2	[55]
97.5Si2.5P (TEP)	270	0.308	4.4	
97.5Si2.5P (H_3_PO_4_)	152	0.235	4.8	
80Si15Ca5P	351	0.49	4.6	[18]
70Si15Ca5P	319	0.49	4.6	
60Si15Ca5P	310	0.43	4.3	
100Si	384	0.4	4.9	[56]
90Si5Ca5P	330	0.35	4.9	
80Si15Ca5P	351	0.36	4.8	
70Si25Ca5P	303	0.33	4.8	
80Si10Ca5P5Fe	260	0.26	3.5	[57]
80Si5Ca5P10Fe	334	0.3	3.6	
80Si0Ca5P15Fe	367	0.36	3.7	
80Si15Ca5P	342	0.38	3.62	[34]
80Si10Ca5P5Mg	274	0.35	3.31	
80Si10Ca5P5Zn	175	0.23	3.33	
80Si10Ca5P5Cu	237	0.31	3.66	
80Si10Ca5P5Sr	247	0.31	3.66	
80Si15Ca5P	515	0.58	4.7	[58]
76.5Si15Ca5P3.5Ce	397	0.38	2.9	
76.5Si15Ca5P3.5Ga	335	0.31	3.8	
80Si15Ca5P	317	0.37	4.1	[59]
80Si10Ca5P5Zr	287	0.32	3.7	
80Si5Ca5P10Zr	278	0.33	4.1	
80Si5P15Zr	277	0.27	3.4	
80Si15Ca5P	265	0.33	5.29	[60]
75Si15Ca5P5B	234	0.24	5.28	
70Si15Ca5P10B	194	0.21	5.09	

**Table 3:** Example of structural parameters of MBG and bioactive glass (BG) scaffolds before and after loading gentamicin drug (Gen) and their drug loading efficiency [73].

Samples	Surface Area (m^2^/g)	Pore Volume (cm^3^/g)	Pore Diameter (nm)	Drug Loading (%)
MBG	334.4	0.348	4.8	-
MBG-Gen	208.9	0.216	4.4	12.33
BG	86.7	0.099	-	-
BG-Gen	53.1	0.081	-	5.03

**Table 4:** Positive effects associated to the release of therapeutic ions from MBGs and main properties.

Therapeutic Ions Released from MBG		Concentration Level (mg/L)	Functional Properties				Ref.
			**Osteogenesis**	**Cementogenesis**	**Angiogenesis**	**Antibacterial Effect**	
Monovalent	Ag^+^	0.014				✓	[75]
	Li^+^	˂17.28	✓	✓			[76]
Divalent	Sr^2+^	˂22	✓				[77]
	Zn^2+^	˂0.75	✓				[78]
	Mg^2+^	˂100	✓	✓			[78]
	Cu^2+^	˂152			✓	✓	[79]
	Co^2+^	˂25			✓		[69]
Trivalent	B^3+^	<50	✓				[69]
